# CHARACTERIZING PROTEIN STRUCTURES IN BETA-AMYLOID TREATED NEURONS WITH LIMITED PROTEOLYSIS-MASS SPECTROMETRY

**DOI:** 10.1093/geroni/igae098.3641

**Published:** 2024-12-31

**Authors:** Kuren Anderson-Steward, Ying Hao, Deyaan Guha, Ziyi Li, Benjamin Jin, Andrew Singleton, Andy Qi

**Affiliations:** Howard University, Bowie, Maryland, United States; National Institutes of Aging, National Institute of Health, Bethesda, Maryland, United States; Brandeis University, Waltham, Massachusetts, United States; National Institutes of Aging, National Institute of Health, Bethesda, Maryland, United States; National Institutes of Aging, National Institute of Health, Bethesda, Maryland, United States; National Institutes of Aging, National Institute of Health, Bethesda, Maryland, United States; National Institutes of Aging, National Institute of Health, Bethesda, Maryland, United States

## Abstract

Limited Proteolysis by Mass Spectrometry (LiP-MS) is a technique used to observe the structural changes of proteins on a proteome-wide scale. Limited proteolysis consists of a specific, or limited, number of proteases performing hydrolyzation on a large portion of proteins. This causes the protease to detect the weak portion of the hydrolyzed protein and cut it out. The technique of limited proteolysis coupled mass spectrometry aims to alleviate the limitations of classic proteomic analysis, such as being unable to detect protein structural alterations on a wide scale and at high throughput. In this experiment, we used iPSC-derived neurons treated with beta-amyloid and performed classic proteomics and LiP-MS in parallel to observe the proteome scale of structural changes caused by beta-amyloid. Through experimentation, it was found that the control group consisted of 180 protein profiles that the beta-amyloid treated group did not possess using the limited proteolysis-mass spectrometry technique. Additionally, various cleavage sites by proteinase K in the beta-amyloid treated neurons compared to the control neurons, as well as multiple molecular pathways, such as neuron projection cytoplasm, were identified.

